# Sensory Preferences Influencing the Likelihood That African American Pregnant Women Will Consume Nutrient-Dense, Anti-Inflammatory Foods

**DOI:** 10.3390/ijerph23080954

**Published:** 2026-07-24

**Authors:** Najjuwah Walden, Lora Iannotti, Gifty Aboagye-Mensah, Rachel G. Tabak

**Affiliations:** 1 Department of Psychiatry, Washington University School of Medicine, St. Louis, MO 63110, USA; 2 Bursky School of Public Health, Washington University in St. Louis, St. Louis, MO 63110, USA

**Keywords:** food acceptability, anti-inflammatory, pregnancy

## Abstract

**Highlights:**

**Public health relevance—How does this work relate to a public health issue?**

- This study assesses what African American pregnant women consider when negotiating the decision to consume select nutrient-dense, anti-inflammatory foods.
- This study demonstrates that food acceptance is established when foods meet taste, texture, appearance, and enjoyment needs.

**Public health significance—Why is this work of significance to public health?**

- Although previous research has established the importance of nutrition in pregnancy, no studies have assessed gastronomic barriers and the facilitators of consuming nutrient-dense foods.
- This study underscores the significance of considering what food options populations will and will not consume when establishing feasible dietary recommendations.

**Public health implications—What are the key implications or messages for practitioners, policy makers and/or researchers in public health?**

- Given the importance of nutrient-dense foods for maternal–fetal health, future research should assess diet change and intake outcomes when dietary recommendations reflect culturally acceptable and preferred foods.
- Future research is needed to assess the implementation potential of dietary recommendations that consider acceptability determinants and those that do not.

**Abstract:**

Understanding how sensory preferences shape the acceptance of nutrient-dense foods is essential for improving prenatal nutrition. This exploratory study examined taste, smell, texture, appearance, and enjoyment factors influencing food acceptability among (n = 21) African American pregnant women, examining how determinants must be met for consumption. We administered surveys to identify preferred foods and analyzed the nutrient quantity of vitamin A, B vitamins, vitamin D, iodine, iron, calcium, magnesium, selenium, and omega-3 fatty acids in each food, given their influence on prenatal health. Nutrient-dense, anti-inflammatory foods were identified by ranking preferred foods by nutrients and selecting those that consistently ranked as top contributors. Focus groups further explored conditions that promote or hinder intake, and content analysis was conducted using NVivo. Preferred nutrient-dense, anti-inflammatory foods included cashews, peanuts, almonds, pistachios, black-eyed peas, pinto beans, salmon, carrots, red bell peppers, and spinach. Participants cited low acceptance due to allergies, undesirable textures (“dry” or “slimy”), unpleasant taste or appearance, a lack of cravings, preference for alternatives, unenjoyable preparation methods, and foods perceived as “display-only.” High acceptance was linked to appealing taste and texture, flexible preparation options, and perceived health benefits. These considerations suggest that taste, texture, appearance, and enjoyment are important considerations for prenatal food intake.

## 1. Introduction

Food acceptability, or liking, refers to the interaction between a food item and the individual consuming it at a specific point in time [1,2]. The determinants of food acceptability are numerous and complex, with some inherent factors being largely immutable [3,4]. Innate attraction to sweet tastes and aversion to bitter flavors tend to remain stable, leading to food preferences derived from likable flavors and other sensory effects [5]. Food acceptance and consumption occur when foods align with an individual’s sensory preferences, including taste, texture, smell, appearance, and overall liking [6,7]. Despite clear evidence that sensory properties shape eating behavior, little is known about how these sensory preferences operate specifically in relation to healthy foods and influence their acceptance and consumption.

Most research on food acceptability has focused on ultraprocessed foods (UPFs), whose sensory profiles are engineered to match or intensify consumer preferences [7]. Industrial additives and processing techniques heighten texture, olfactory, and visual appeal, while refined sugars, salts, and fats meet consumer demands for taste [8]. UPFs are high in flavor-enhancing additives but typically low in essential vitamins, minerals, and omega-3 fatty acids [9]. The consequences of inadequate nutrient intake are the most detrimental in pregnancy and include perinatal depression, preeclampsia, hemorrhage, low birthweight, preterm delivery, and fetal abnormalities [10,11,12]. Despite the risk of pregnancy complications, sensory attributes drive the consumption of UPFs [13]. However, the strategies needed to make foods associated with improved maternal–fetal health meet consumer expectations for sensory pleasure remain underexplored.

The nutrients needed to minimize the risk of compromised maternal–fetal health include vitamin B12, calcium, vitamin D, folate, iodine, iron, magnesium, and *n-3* polyunsaturated fatty acids (PUFAs; omega-3) [11]. Low calcium, magnesium, vitamin D, and iron levels are associated with hypertensive disorders, preeclampsia, and a higher risk of preterm labor and preterm birth [11,14,15,16,17]. Low B12, iron, magnesium, and calcium are also linked to fetal growth restriction and low birthweight [11,18,19,20,21]. Deficiencies in folate and B12 increase the risk of neural tube defects, while inadequate iodine and omega-3 intake impairs fetal brain development and cognition [11,22,23,24,25]. Vitamin B12 deficiency increases miscarriage risk, and low calcium contributes to maternal musculoskeletal symptoms, including cramps and tremors [11,19,26]. The inadequate intake of vitamins A, D, and B (thiamin, riboflavin, niacin, pantothenic acid, B6, folate, and B12); iodine; iron; selenium; PUFA 20:5 n-3 (EPA); and PUFA 22:6 n-3 (DHA) is also associated with perinatal depression [27,28,29,30,31,32,33,34,35]. But these nutrients can be found in anti-inflammatory foods [36,37,38,39,40,41,42,43,44,45,46,47].

Anti-inflammatory foods include fatty fish (salmon, mackerel), yogurt and kefir, nuts and seeds, legumes, unrefined grains, herbs and spices, olive oil, fruits, and vegetables [48,49]. Poor or inconsistent dark green leafy vegetable intake is associated with a two-fold increase in the risk of pregnancy complications [50]. However, adherence to dietary recommendations for vegetables is reportedly as low as 0% in pregnant women in the U.S. [51]. Moreover, across all racial and ethnic identities, the prevalence of inadequate vegetable intake is the highest among African American pregnant women, while the incidence of nutrition-associated pregnancy complications remains the highest in this group, even when controlling for socioeconomic status [52,53]. This disparity underscores the need to understand not only structural barriers to healthy eating but also the sensory preferences and acceptability factors that shape daily food choices. Understanding these determinants is particularly important for identifying acceptable strategies to prevent nutrition-related pregnancy complications.

The purpose of this study is to characterize the determinants of nutrient-dense, anti-inflammatory food acceptability in pregnancy among African American pregnant women. Building on the World Health Organization (WHO) definition of food acceptability, which encompasses sensory attributes, cultural preferences, and personal beliefs [54], this study aims to examine how these determinants shape real-world consumption patterns in pregnancy among African American women. The study objectives include identifying the nutrient-dense, anti-inflammatory foods preferred during pregnancy; evaluating how sensory factors influence the acceptance of each food; and assessing the beliefs that guide food choices. Together, these aims support a central inquiry into whether one or more determinants of acceptability must be satisfied for a food to be consumed, and we hypothesize that interaction among these determinants shapes dietary behavior. The results have significant implications for identifying nutrient-dense, anti-inflammatory foods to pilot with African American pregnant women and understanding how recommendations should be designed.

## 2. Materials and Methods

### 2.1. Conceptual Framework

Food acceptability described in relation to the five elements of palatability—taste, smell, texture, appearance, and enjoyment [55,56]—was used as a conceptual framework to guide data analysis. Taste refers to the profiles of food, including but not limited to sweetness, saltiness, and fattiness [57]; smell refers to food odors in relation to appetite stimulation [58]; texture refers to the particles in food detected by touch, sight, hearing and kinesthetic senses [59]; appearance refers to visual cues, including proximity, visibility, color, variety, portion size, height, shape, number, volume, and surface area [60]; and enjoyment refers to favorable associations between eating pleasure and dietary outcomes [61]. An “acceptability” conceptual framework is a structured approach used in previous research with African Americans to understand and analyze the factors that influence willingness to accept and consume a particular food [62,63].

### 2.2. Study Design and Participants

This exploratory qualitative study was designed in partnership with the YWCA of Metro St. Louis. The YWCA conducted nutrition education classes during their Early Head Start (EHS) program and invited the PI to collect data during these classes from January to February 2024. This study aimed to identify nutrient-dense foods preferred by African American pregnant women and the conditions that determined food acceptability. The Washington University in St. Louis Institutional Review Board granted ethics approval ahead of study activities (IRB # 2023-12155), and all participants provided written informed consent. The study population, as reported by site administrators prior to data collection, was African American, 18 to 25 years of age, and pregnant or parenting newborns less than six weeks old. EHS serves low-income families, with eligibility generally capped at 100% of the federal poverty guidelines, and families experiencing homelessness, children in foster care, and those receiving public assistance are eligible regardless of income. Additional identifying information was not collected from participants during data collection. During EHS classes, the PI presented this study and information on how to participate. A sample of 21 participants completed surveys and focus group interviews.

### 2.3. Data Collection

This study surveyed preferred foods from a self-administered REDCap (https://project-redcap.org/, accessed on 21 July 2026) survey completed by participants on their smartphones during the first nutrition education class. Open-ended, self-administered dietary surveys are validated across maternal nutrition, sensory science, and community-based nutrition interventions as reliable tools for capturing culturally specific preferred foods [64,65,66]. The survey included the following open-ended questions:

1. Imagine you’ve just walked into the store for food. What do you shop for first?
2. What is your favorite food?
3. What foods do you buy often?
4. What foods do you wish you ate more?
5. What foods would you eat every day?
6. What [food group (i.e., vegetables)] do you like to eat?

This study surveyed the fruits, vegetables, proteins, grains, dairy, and nuts participants eat due to the concentration of nutrients associated with maternal–fetal health—vitamins A, D, and B (thiamin, riboflavin, niacin, pantothenic acid, B6, folate, and B12); iodine; iron; selenium; calcium; magnesium; EPA; and DHA—within these foods [11,12]. Two analysts (NW and GAM) conducted a content analysis of fruits, vegetables, proteins, grains, dairy, and nuts listed in open-ended survey responses using NVivo 14 (https://lumivero.com/products/nvivo/, accessed on 21 July 2026) to identify participant-preferred foods. All responses are described (Table 1).

All foods listed by participants were analyzed using FoodData Central (https://fdc.nal.usda.gov, accessed on 21 July 2026) to identify the quantity of vitamins A, thiamin, riboflavin, niacin, pantothenic acid, folate, B12, D, iodine, iron, calcium, magnesium, selenium, EPA, and DHA within each food. FoodData Central converts foods and beverages into gram amounts and determines their nutrient values [67]. Nutrient compositions were reported in a matrix in Microsoft Excel (Microsoft Corporation, Version 16.0), with rows representing nutrients and columns representing foods. Nutrient-dense foods were identified based on a nutrient profiling approach. Nutrient profiling is a systematic approach to assessing a food’s nutritional value by ranking its nutrient content per reference amount (e.g., per 100 g) [68]. This study identified anti-inflammatory foods—fatty fish (salmon, mackerel), yogurt and kefir, nuts and seeds, legumes, unrefined grains, fruits, and vegetables [48,49]—that appeared at the top of ranked lists in the matrix for at least one or more nutrients of interest (Table 2). This study selected foods after identifying a combined intake that would meet the criteria for high nutrient intake, defined as >20% by the U.S. Food and Drug Administration [69]. The resulting selection included: raw peanuts, raw almonds, raw pistachios, dried black-eyed peas, dried pinto beans, baked or broiled salmon, cooked carrots, raw red bell peppers, and raw spinach. Although the vitamin B5 content in preferred foods was not considered high, the most nutrient-dense sources defined by the National Institute of Health Office of Dietary Supplements were not listed as preferred foods by study participants.

After identifying preferred nutrient-dense, anti-inflammatory foods, NW conducted a focus group during the second nutrition education class with study participants to examine the conditions that determined consumption. The foods highlighted during the focus group included peanuts, almonds, pistachios, black-eyed peas, pinto beans, salmon, carrots, red bell peppers, and spinach. Each food was discussed one at a time, clustered by food group (i.e., nuts, legumes, salmon, and vegetables) and with the same sequential questions:

1. What would you say if someone told you [insert food] are good for you?
2. How do you feel about eating [insert food] every single day?
3. How easy would it be to eat [insert food] every single day?
4. Is there anything you could do that would make it easier to eat [insert food]?
5. If you had to eat [insert food] everyday, how would you want to eat them?

A handout displaying an image of the food in its nutritionally adequate serving size photographed in color on a white dinner plate was provided to each participant prior to the question sequence. Additional follow-up questions were facilitated until participants did not provide new responses but repeated what was previously said. The audio-recorded focus group discussion lasted 90 min and was facilitated by NW, an African American cis-gendered woman with experience in focus group data collection. NW introduced herself to the participants as a birth doula present to discuss prenatal and postpartum nutrition. NW had no prior relationship with the participants.

### 2.4. Data Analysis

This study transcribed the audio recordings of the focus group to conduct a content analysis of responses to focus group questions using NVivo 14 (https://lumivero.com/products/nvivo/, accessed on 21 July 2026). Data were transcribed by Rev (https://rev.com, accessed on 21 July 2026) and corrected by the PI for the correctness of African American Vernacular English and slang terminology. Two analysts (NW and GAM) conducted the content analysis. To ensure analytic rigor, inter-rater reliability was assessed following independent first- and second-level coding. Coders compared code applications and achieved 100% agreement through iterative discussion, refining the codebook as needed to improve clarity and consistency. Discrepancies were resolved using a structured adjudication process: NW and GA first attempted resolution through discussion; unresolved disagreements were reviewed by a support specialist at the Washington University Writing Center. First-level coding organized participant responses at the question level. Second-level coding involved re-categorizing responses. For example, a participant may have mentioned why a food would not be consumed when asked how easy it is to eat a particular food daily. This response was coded as reason for low acceptability. Third-level coding includes generating a list of responses coded for taste, smell, texture, appearance, and enjoyment. The analysis describes the conditions that determined the acceptability of discussed foods.

## 3. Results

Participants described the taste, texture, appearance, and enjoyment of foods associated with food acceptability and lack thereof. However, no participants described the odor profiles of food as a determinant of acceptability. The taste of carrots, nuts, and legumes was important when considering consumption. The texture of carrots and red bell peppers determined intake. The appearance of peanuts, salmon, and spinach was important when participants considered eating these foods. Enjoyment was also important when considering why peanuts, almonds, salmon, black-eyed peas, and pinto beans were acceptable.

### 3.1. Peanuts

Participants described peanuts as a food they accepted but conditionally. Due to dryness, allergies, dislike, and perceived inability to remember to eat peanuts, participants believed peanuts were a food they would eat but not every day. When asked how easy it would be to eat peanuts every day, participants (n = 3) voiced “it would not be easy.” One participant explained:

“Because they get all in between your teeth. I keep doing this all day.” *Makes gesture sucking teeth to remove peanuts.* “I don’t buy them at all. But if they come for free, I’m going to eat me some. It would be like a last resort type.”

(Taylor, Participant 3)

When asked how peanuts could be made more desirable, participants responded with “sugar, cinnamon, and butter;” salt; no salt; and honey-roasted. The ways peanuts could be prepared to make them more desirable included “in noodles,” “in trail mix,” “inside M&Ms,” and “sometimes peanut butter.” All participants agreed that peanut butter is better than peanuts. One participant explained, “Because it’s creamy. It don’t get all up in the teeth.” However, participants admitted they would not eat peanut butter every day—every other day at most and every day if food, like cereal, were “peanut-flavored.”

### 3.2. Almonds

Among participants who were not allergic to almonds, a few (n = 3) described this nutrient-dense, anti-inflammatory food as one that they like. However, the taste and toughness of almonds were justifications for not liking almonds. As described by one participant, “they’re hard and it gets between your teeth.” Whereas this participant also believed almonds “taste funny,” others enjoyed almonds “on ice cream” and “with some sugar and cinnamon.” One participant explained the following:

“I would really consider eating [almonds] with any product that’s pertaining to dairy. Just because it can balance out the texture of it versus you just eating some [almonds] or something and then drinking something afterwards. It’ll kind of leave an aftertaste in your mouth. So, I would just consider it with a dairy product [like ice cream].”

(Devin, Participant 2)

Additionally, participants wanted to eat almonds in cereal, in yogurt, and dipped in chocolate. Participants also reported liking almond milk, with one participant liking almonds only if they are in almond milk. Participants (n = 4) not allergic to almonds reported that drinking almond milk to get your almonds every day would be a “piece of cake.”

### 3.3. Pistachios

Participants (n = 4) believed that pistachios were “good” and liked them enough to eat them every day but only when prepared using a specific method. For example, “as long as they have the sea salt” and them being in oatmeal or pistachio ice cream were specified as ways that they would eat pistachios every day. One participant explained, “I would most likely put it in oatmeal just because I know oatmeal is a good grain for when you’re breastfeeding, so I would definitely recommend that inside of oatmeal.” However, distaste towards eating pistachios every day was attributed to having to “work for my food” and bland taste. Although participants agreed that sea salt improved the taste of pistachios, participants were unwilling to buy pistachios for everyday consumption because “I’m going to go get me some pistachios” is not a thought participants have when shopping for food.

### 3.4. Black-Eyed Peas

Participant acceptability towards black-eyed peas varied from “not in the house, not in the mouth” to “I love black-eyed peas.” Reasons for not liking black-eyed peas include texture and companion foods commonly used when preparing black-eyed peas. One participant describes, “You all can keep those. They nasty, they pasty, they slimy, people put okra in them. I don’t know why they do that.” However, alternative preparation methods were more acceptable, including black-eyed peas made with neck bones and black-eyed peas made with sugar or brown sugar. One participant explains the following:

“Honestly, to me, they don’t really have a specific taste. They really taste like… Not baked beans, but any other beans that you can cook inside of a pot with a certain type of meat. So, once you cook them down, they don’t really have a taste to them. I think it’s just a texture or just the word ‘black-eyed’ on it that gets people worked up. But they honestly taste pretty good to me, mixed with meat of course. I’ve never had it separately, but everybody tastes bud is different. So, I would definitely eat that every day if I had to, with meat.”

(Alex, Participant 4)

Participants (n = 4) admitted to being willing to eat black-eyed peas every day, “almost” every day, and on holidays. Participants desiring to eat black-eyed peas less often explained that having to cook black-eyed peas is difficult, and eating them every day would be “boring.” However, participants suggested that “the side would probably make it better,” using “cornbread” as an example.

### 3.5. Pinto Beans

Participants reported eating pinto beans, but participants (n = 3) did not like them enough to eat them every day. There were two specific preparation methods participants preferred when considering consuming pinto beans every day. One participant described the following:

“Put some smoked turkey tails or you put you some ham hock or a ham shank in them beans. There’s a whole bunch of butter. Put a little sugar in it for the kids—only on their bowl, though, I don’t want the sugar—with some cornbread and wooh.”

(Taylor, Participant 3)

Meanwhile, another participant described, “I just ain’t gon’ want them beans. I like to eat them in Chipotle—like in Mexican food with like rice.” However, for other participants (n = 3), pinto beans were not a food they would eat every day. One participant said “I would get tired” when explaining why it would be difficult to eat pinto beans every day, while others explained their preference for other beans. Participants explained “I’m a chili bean only person” and “I’m more of a black bean person” when explaining how they felt regarding pinto beans. These participants concluded, “they good, but I wouldn’t eat them every day.”

### 3.6. Salmon

Participant attitudes towards salmon ranged from “I’m good” (n = 2) to “love” (n = 2). Reasons for not wanting salmon included not having “a taste for it” and liking other fish, including “fried fish” and “tilapia, swai, and shrimp.” Household allergies also affected salmon intake frequency and likeness. As one participant noted, “My brother’s allergic to fish, so we don’t eat it that often. So, once you go without it, you don’t really need to eat it all the time.” However, having a shellfish allergy was a justification for eating salmon. One participant described, “I like salmon, but that’s cause I can’t have shellfish. So, the main thing I would eat is salmon.”

Most participants (n = 4) liked that salmon could be prepared in many ways. Participants reported eating salmon in croquettes, sushi, rice, salad, pasta, and alfredo. Preferred preparation methods involved grilling, frying, searing, smoking, and Cajun seasoning. Participants believed it would be “pretty easy” to eat salmon every day if it is made in different ways and in different dishes. However, participants were dissatisfied with croquettes every day: “Because who going to eat salmon croquettes every day? Probably old people with hot water cornbread.”

### 3.7. Carrots

Participants reported liking carrots but with exceptions. One participant did not like carrots cooked, while another did not like carrots that were hard or raw. One participant reported liking carrots only in a pot roast: “Put it in a pot roast. Carrots taste just like the pot roast. Soft and they not hard. That’s it.” However, participants reported liking carrots many ways, including sweet, unsweet, salt and peppered, with ranch, and in salads. Participants also reported liking them as a snack. Participants believed it would be easy to eat carrots every day because they can be eaten “throughout the day” and “in different dishes.” However, one participant believed she could only eat carrots every day “on a diet.”

### 3.8. Red Bell Peppers

Participants reported liking red bell pepper in many ways. Some ate red bell pepper raw (n = 1), seared (n = 1), or stuffed (n = 1), while others preferred red bell pepper in chili, stuffed, or in fajitas, hamburgers, meatloaf, chicken, baked pork chops, or “everything.” As one participant described, having to eat red bell pepper raw would hinder their ability to eat red bell pepper every day. However, participants believed it would be “very easy” to eat red bell pepper every day if it was “cooked” and prepared “in many ways.” As one participant described, “I would have to find different ways to eat it because I probably couldn’t just eat them by they self every day.”

### 3.9. Spinach

Impressions of spinach ranged from “ew” to “I would eat spinach every day.” Reasons why participants do not like spinach included its taste. Participants preferred spinach in a dip, sandwich, salad, or smoothie. While some participants (n = 3) believed they “probably could” eat spinach every day, one participant believed they would not, due to being averse to repetition in their diet. However, some participants (n = 2) explained that if spinach is “overpowered by something else” and they “can’t taste it,” they could eat spinach every day. One participant explained the following:

“When it comes to spinach, I’m not too much of a spinach fan. The only time I’ll eat spinach is if it’s a spinach Alfredo pizza because I can’t taste it. I don’t like to taste of it. If I could see it, because you can see it on Alfredo pizza, that’s fine. But it looks more like lettuce on the Alfredo pizza. So, if it doesn’t look like it’s been grilled and shriveled up, then I’m not going to try it. If it looks like a full-blown leaf and it’s not lettuce, I’m not going to eat it.”

(Jordan, Participant 1)

## 4. Discussion

The current exploratory study aimed to evaluate the sensory preferences that influence the acceptability and consumption of nutrient-dense, anti-inflammatory foods in a sample of African American pregnant women. The results suggest that preferred foods are acceptable and consumed if prepared in ways that meet sensory preferences in noniterative ways. Participants discussed the acceptability of select nutrient-dense, anti-inflammatory foods they preferred, including peanuts, almonds, pistachios, black-eyed peas, pinto beans, salmon, carrots, red bell peppers, and spinach. The researcher asked directly about these anti-inflammatory foods because of their connection to vitamins A, D, and B (thiamin, riboflavin, niacin, pantothenic acid, B6, folate, and B12); iodine; iron; selenium; calcium; magnesium; and n-3 polyunsaturated fatty acids associated with minimizing the risk of perinatal depression, hypertensive disorders, preeclampsia, preterm labor and birth, fetal growth restriction, low birthweight, neural tube defects, impaired fetal brain development and cognition, miscarriage, and maternal musculoskeletal symptoms [11,12]. Participants described taste, texture, appearance, and enjoyment as determinants of food acceptability. However, no participant described smell as a food acceptability determinant. Therefore, taste, texture, appearance, and enjoyment may be important to consider when developing strategies to increase nutrient-dense, anti-inflammatory food intake among African American pregnant women.

### 4.1. Taste

Regarding taste, participants most often preferred sweet, salty, and umami flavors. Prior research indicates that sweetness preferences are strongly associated with liking foods containing added sugars [70], and participants’ descriptions of “sweet” frequently included cinnamon—a spice whose flavor is detected through sweet-associated pathways [71,72]. To enhance flavor, participants also described using salt, butter, and meat. Although some individuals have a genetic predisposition toward sweet or salty tastes [73,74], individuals can also exhibit a preference for savory flavors [75,76]. This may explain why meat and butter were cited as ingredients that make foods more palatable [77,78]. For some participants, this palatability was driven by the texture contributed by added fats, while for others it was tied to aroma [77,79]. Participants also referenced Cajun seasoning blends emblematic of broader African American cooking traditions [80], reflecting a preference for a flavor profile. Collectively, these findings suggest that preparation methods for nutrient-dense, anti-inflammatory foods that align with preferred sweet, salty, and umami flavor profiles may support greater intake. However, additional research is need to determine whether educating pregnant women on foods that meet their taste preferences contributes to improvements in dietary intake.

### 4.2. Texture

The consumption of nutrient-dense, anti-inflammatory foods was also dependent on food texture. Some participants were averse to dry, hard textures, with particles that got in between their teeth, while others preferred raw carrots and red bell peppers because of their texture. A preference for hard foods is common among adults due to the sensory experience of crunch [81]. However, a dislike for audible chewing or an inability to chew hard foods can lead to a dislike for foods that crunch [82,83]. While participants who did not prefer hard foods did not mention crunch as a factor, consuming nuts with milk or ice cream was a listed strategy to make nuts more palatable. The liking of these complementary foods may be due to the effects they have on texture or the effects that they have on the ability to hear or feel “crunch.” Additional research is needed to evaluate the desired effects of complementary foods.

Similarly, participants reported a dislike for foods that resembled “slime” or “paste.” Like having a preference for hard foods, a strong dislike for slimy foods is a common sensory experience, often explained by sensory processing difficulties or associating gooey textures with spoilage or toxicity [84]. Therefore, texture preferences determine food preparation methods. However, the intake of nutrient-dense, anti-inflammatory foods may increase if dietary recommendations accommodate desired sensory experiences. The previous literature has found that textural improvements are associated with nutritional improvements in aging populations [85]. However, further research is warranted to determine whether providing pregnant women with guidance on foods that align with their textural preferences translates into measurable improvements in dietary patterns.

### 4.3. Appearance

In addition to taste and texture, appearance was an important food acceptability determinant mentioned by participants. For example, participants mentioned consuming peanuts if they were displayed for free or an ingredient in a meal they were eating. The phenomenon of people eating foods that are free but that they would not buy is explained by previous research as being due to a relationship between a stimulus and reward [86]. Thus, the presence of a free food may trigger an associative memory that it should be eaten, even if it is not desired. However, the acceptance of peanuts as an ingredient within a meal may alternatively suggest that participants did not perceive peanuts as a food that should be eaten alone. Therefore, low intake may suggest that few meals include peanuts.

In addition to accepting food if it was free or inside a meal, participants reported only accepting foods if they were prepared a certain way. The desired appearance of spinach was limited to “shriveled up” instead of a “full-blown leaf.” Therefore, food preparation methods determined the acceptability of nutrient-dense, anti-inflammatory foods. However, additional research is need to determine whether the inclusion of these foods in more meals, prepared using preferred methods, may lead to an increase in food intake.

### 4.4. Enjoyment

Participants remembered the nutrient-dense, anti-inflammatory foods they did and did not consume and whether they enjoyed them. Enjoyment determined the acceptability of peanuts, pistachios, and salmon. However, some participants could not consume these foods due to allergies. Living in a household with someone who had a food allergy led to a limited or absent intake of these foods, even if the allergy was only present in childhood. Therefore, household eating habits in childhood may contribute to nutrition insecurity in adulthood [87]. Adults who do not have allergies may adopt the eating habits of others who do [88]. This may not only contribute to limited food acceptability but may illustrate that eating habits are established at the household level, and one individual’s vulnerability to consuming too little of a food due to an allergy may contribute to your own, even if you do not have that allergy, by virtue of living in the same household [88,89,90]. However, limited enjoyment also limited food intake if foods were too difficult to prepare or consume. Participants reported a limited acceptability of pistachios because “I don’t work for my foods.” Participants also believed eating black-eyed peas was difficult because cooking them was difficult, whereas eggs were enjoyed because they were “easy.” However, participants reported enjoying legumes because they were often prepared with animal protein or in Mexican food. Therefore, when developing recommendations for how to prepare nutrient-dense, anti-inflammatory foods, researchers should consider allergies, how to prepare foods that are being explored for the first time, and preparation methods that are easy. In addition, increasing opportunities to prepare foods at home that are enjoyed at restaurants may also increase the uptake of dietary recommendations and intake of nutrient-dense, anti-inflammatory foods.

The results suggest that one or more determinants of food acceptability are considered when deciding to consume nutrient-dense, anti-inflammatory foods. However, enjoyment was the most frequently cited determinant of consumption, then appearance, taste, and texture. Defined as a favorable association between eating pleasure and dietary outcomes [61], this highly cited determinant suggests that women are likely to consume foods they remember liking in the past. Adopting increased nutrient-dense, anti-inflammatory food intake may be the most successful when recommending foods African American pregnant women already consume and enjoy consuming, rather than introducing new foods. Additional research also suggests that whether or not a recommended food is already consumed is the strongest determinant of dietary recommendation acceptance [91]. Therefore, the current study highlights foods that may contribute to increased dark green leafy and red-orange vegetable, nut, legume, and fish choices in African American pregnant women.

### 4.5. Strengths and Limitations

A significant strength of this study is its ability to explore sensory attributes, cultural preferences, and personal beliefs associated with nutrient-dense, anti-inflammatory food intake among African American pregnant women. These are determinants of food acceptability defined by the WHO [54], but rarely are they studied among African Americans. Alternatively, prior research concludes that “healthy” foods are not accessible or affordable among African Americans due to a combination of factors, including economic inequality, the prevalence of food deserts, and historical and ongoing disinvestment in African communities, and thus socioeconomics has been a dominant focus in dietary research on African Americans [92]. However, no participant mentioned cost or price as a barrier that would need to be removed to consume nutrient-dense, anti-inflammatory foods. Participants may not perceive cost barriers associated with purchasing peanuts, almonds, pistachios, black-eyed peas, pinto beans, salmon, carrots, red bell peppers, or spinach. Research conducted by the United States Department of Agriculture (USDA) has also identified that the cost per weight of healthy foods is less than that of less healthy foods [93]. However, the interview instrument did not include cost-related questions, and their inclusion may have yielded participant perspectives on the affordability of discussed foods. Instead, this study highlights that African American pregnant people have taste, texture, appearance, and enjoyment needs that need to be obliged to consume nutrient-dense, anti-inflammatory foods.

Although, there is a general perception that eating “healthfully” is in opposition to African American culture and to do so means giving up on food taste and cultural symbolism [94], this study identified methods for preparing healthy foods that are specific to African American people. By increasing opportunities to consume preferred nutrient-dense, anti-inflammatory foods in ways that are acceptable, we may also observe an increase in intake and maternal–fetal health. While poor or inconsistent dark green leafy vegetable intake is prevalent in pregnancy [50], recommending spinach “in a dip, sandwich, or smoothie” may increase the acceptability of dietary recommendations and consumption in populations with low intake. Pregnant women who consume more nuts and seeds also show an increased likelihood of having infants with higher cognitive scores [95]. Thus, recommending “almonds paired with dairy,” “pistachios with oatmeal,” and peanut butter, as discussed in this study, may also provide references for integrating more nut intake. Higher omega-3 and salmon intake helps support fetal brain development and cognition [11,22,23,24,25], while folate-rich legume intake supports women in lowering their risk of gestational diabetes and depression [29,96], and the methods of preparing these proteins mentioned in this study may support women with increasing their consumption. The prenatal consumption of red-orange vegetables, including carrots, is also tied to an increased likelihood of infant acceptance when transitioning to table foods [97]. Therefore, integrating acceptability determinants is an important consideration for dietary recommendations that may yield short-term and long-term benefits.

However, the results of this study are not generalizable. The study design is exploratory and did not aim to provide a basis for broader conclusions. This study was conducted with a convenience sample of African America pregnant women participating in the Early Head Start program at the YWCA of Metro St. Louis, providing a limited geographic and demographic representation of considerations. Without collecting data on gestational age, obstetric history, and additional clinical information, this study is limited in its ability to generalize the findings to women with similar health histories. The study results also cannot be generalized to other pregnant populations, including African American women with dietary habits influenced by alternative cultural and environmental factors, such as immigration and acculturation or higher income and greater food access.

Although this study conducted an in-depth 90 min focus group, it did not conduct multiple focus groups due to time constraints within the allotted nutrition education class, leaving many perspectives and experiences undocumented. The responses provided by participants may also be subject to social desirability bias. Participants may have provided responses consistent with researcher expectations. Recall bias may also affect the preferred foods participants listed. Evaluating current dietary intake using food frequency questionnaires or 24 h dietary recall may have identified additional foods participants prefer but did not list. Future research should expand representation to African American pregnant women who are older; middle-to-high-income; residing in rural, semi-rural, and semi-urban geographies; and in varying stages of pregnancy to expand knowledge on acceptability determinants, including smell. Pregnant women experience heightened smell sensitivity, or hyperosmia [98], and including more women in their first and second trimesters in research may yield additional determinants not identified by this study. Additionally, food acceptability was evaluated while viewing 100 g servings of select foods to standardize comparisons, and this may exceed typical serving sizes. Therefore, study results may vary when considering the serving sizes participants typically consume.

### 4.6. Implications

Although prior research suggests that a food acceptability conceptual framework increases the understanding of factors that influence acceptance and consumption, future research is needed to understand whether integrating important considerations for food acceptability within dietary recommendations leads to adoption and whether adoption is also associated within changes in maternal–fetal health outcomes in African Americans. Prenatal nutrition education programs that integrate the study findings may improve the desirability of nutrient-dense, anti-inflammatory foods among African American populations. Culinary nutrition education that discusses the importance of select foods while preparing them in preferred ways may also increase the sensory appeal of nutrient-dense, anti-inflammatory eating, as evidenced in the previous literature [99]. Nutrition education counselors, including employees of the Special Supplemental Nutrition Program for Women, Infants, and Children (WIC) program, may also benefit from training on using taste, texture, enjoyment, and appearance preferences to increase the consumption of clinically beneficial foods. Therefore, further research is needed to identify the clinical and implementation outcomes of integrating taste, texture, enjoyment, and appearance preferences into dietary recommendations.

## 5. Conclusions

In conclusion, this study offered important insights for interpreting what vegetables, nuts, legumes, eggs, and fish are acceptable and why. The participants highlighted the need for food to taste sweet, salty, and umami; for texture to accommodate desired sensory experiences; for foods to be prepared as ingredients within meals made at home; and for foods made at home to resemble foods eaten at restaurants. When individuals find foods acceptable, they are more likely to adopt a dietary pattern that includes them [100,101,102]. Therefore, further research is necessary to determine whether integrating considerations for food acceptability within dietary recommendations benefits implementation and clinical outcomes.

## Figures and Tables

**Table 1:** Food preferences categorized by food group.

Food Group	Preferences
Fruits	grapes, apples, oranges, bananas, mangos, pineapples, strawberries, blueberries, watermelon
Vegetables	cucumbers, lettuce, spinach, broccoli, Brussels sprouts, green beans, peas, green bell pepper, red onion, red bell pepper, tomato, carrots, orange bell pepper, yellow bell pepper, squash, corn, potatoes, onion
Protein	chicken, bacon, eggs, turkey, beans (black beans, red beans, lima beans, pinto beans, black-eyed peas), fish (cod, salmon, jack/pacific whiting, catfish, tilapia, swai), chili beans
Grains	pasta (spaghetti, fettuccini, manicotti, lasagna, penne), rice (white, jasmine, basmati), oats, cereal, bread (honey wheat, white, cinnamon raisin), pie crust, Hawaiian rolls, garlic bread, cookies
Dairy	milk, alfredo, cheddar cheese
Nuts	peanuts, almonds, pecans, pistachios, cashews, walnuts

**Table 2:** Nutrient content per 100 g serving of preferred anti-inflammatory foods with highest nutrient values.

	A	B1	B2	B3	B5	B6	B9	B12	D	Ca	Fe	Mg	Se	EPA	DHA
Carrots	812	0.1	0.1	0.9	0.2	0.1	16	0	0	31	0.3	12	0.1	0	0
Peppers	157	0.1	0.1	1	.	0.3	47	.	.	6	0.4	11	0.1	.	.
Spinach	283	0.1	0.2	0.6	.	0.2	116	0	0	68	1.3	93	0	0	0
Almonds	0	0.2	1.1	3.6	0.2	0.1	44	0	0	254	3.7	258	4.1	0	0
Peanuts	0	0.6	0.1	12.1	.	0.3	240	0	0	92	4.6	168	7.2	0	0
Pistachios	13	0.7	0.2	1.4	.	1.1	51	0	0	107	4	109	10	0	0
Black-eyed	0	.	.	.	.	.	.	.	.	133	6.8	.	.	.	.
Pintos	0	0.2	0.1	0.3	.	0.2	162	0	0	46	2.1	50	6.2	0	0
Salmon	75	0.2	0.2	10.6	.	0.7	23	3.9	13.7	11	0.4	34	32.1	860	1130
RDA%	103%	150%	133%	179%	6%	160%	140%	138%	91%	75%	262%	204%	85%	860%	565%

Iodine is not included in this table due its absence in selected foods. Vitamins A, folate (B9), cobalamin (B12), D, and selenium (Se) are expressed in mcg units. Thiamin (B1), riboflavin (B2), niacin (B3), pantothenic acid (B5), pyridoxal phosphate (B6), calcium (Ca), iron (Fe), magnesium (Mg), EPA, and DHA are expressed in mg units. “.” indicates that this nutrient quantity is not reported by the USDA FoodData Central database.

## Data Availability

The original contributions presented in this study are included in the article; further inquiries can be directed to the corresponding authors.

## Abbreviations

The following abbreviations are used in this manuscript:

EPA	Eicosapentaenoic Acid
DHA	Docosahexaenoic Acid
PUFA	Polyunsaturated Fatty Acid
UPF	Ultraprocessed Food
